# The ripening disorder berry shrivel affects anthocyanin biosynthesis and sugar metabolism in Zweigelt grape berries

**DOI:** 10.1007/s00425-017-2795-4

**Published:** 2017-10-26

**Authors:** Michaela Griesser, Sara Crespo Martinez, Markus W. Eitle, Benedikt Warth, Christelle M. Andre, Rainer Schuhmacher, Astrid Forneck

**Affiliations:** 1 Division of Viticulture and Pomology, Department of Crop Sciences, University of Natural Resources and Life Sciences, Vienna, Konrad Lorenz Straße 24, Tulln, 3430 Vienna, Austria; 2 Department of Environmental Research and Innovation, Luxembourg Institute of Science and Technology, Avenue des Hauts-Fourneaux, L-4362 Esch/Alzette, Luxembourg; 3 Center for Analytical Chemistry, Department of Agrobiotechnology (IFA-Tulln), University of Natural Resources and Life Sciences, Vienna, Konrad Lorenz Straße 20, Tulln, 3430 Vienna, Austria; 4 Present Address: Department of Food Chemistry and Toxicology, University of Vienna, Währingerstraße 38, 1090 Vienna, Austria

**Keywords:** Anthocyanins, Berry shrivel, Hexose transport, Invertase, Sink establishment

## Abstract

**Timescale analyses suggest the berry shrivel (BS) disorder is induced before veraison with strong effects on anthocyanin biosynthesis, and minor effects on sugar transport and metabolism.**

Berry shrivel (BS)-affected grapes have low sugar contents, high acidity, less anthocyanins and flaccid berries. To date no pathogenic causes are known, and studies to elucidate the molecular basis leading to symptom induction and development are limited. Here we present a study on pre-symptomatic as well as symptomatic BS berries to characterize early metabolic changes, with focus on anthocyanin biosynthesis and sugars metabolism. Healthy and BS berries from six sampling time points were used (BBCH79–BBCH89). Our objectives are (1) to search for the beginning of BS-related physiological processes; (2) to search for key enzymes and sugar transporters involved in BS induction and development and (3) to understand the consequences on polyphenol biosynthesis. We employed high performance anion exchange chromatography coupled with pulsed amperometric detection (HPAEC-PAD) and liquid chromatography–tandem mass spectrometry (LC–MS/MS) technology for sugar and polyphenol analyses, respectively. Additionally we conducted expression analyses (qPCR) of key genes and enzymatic activity assays. Our results show that BS-related processes start before veraison, as determined by slightly reduced hexose contents and reduced expression levels of a vacuolar invertase (*VviGIN1*), two monosaccharide transporters (*VviTMT2*, *VviTMT3*) and the anthocyanin biosynthesis (*VviUFGT*, *VviMYBA1/2*) genes. Lower amounts of delphinidin and cyanidin glycosidic forms were determined, while caftaric acid, quercetin-3-*O*-glucuronide and (+)-catechin were increased in BS berries. Although not all results were conclusive, especially for the sugar metabolism, our data provide important knowledge to improve the understanding of the highly complex berry shrivel ripening disorder.

**Electronic supplementary material:**

The online version of this article (doi:10.1007/s00425-017-2795-4) contains supplementary material, which is available to authorized users.

## Introduction

Grape berry growth and ripening is a process following a double sigmoid developmental curve (Coombe 1992; Conde et al. 2006). The complex morphological and physiological processes taking place during ripening can be disturbed leading to physiological ripening disorders (or shrivel disorders) such as bunch stem necrosis (BSN), late-season dehydration and berry shrivel (BS), also named sugar accumulation disorder (SAD) (Krasnow et al. 2009a) or suppression of uniform ripening (SOUR) (Bondada 2014). Although these shrivel disorders lead to the same final symptom, shriveled berries, they are distinct in causes, development processes and speed (Bondada and Keller 2012; Griesser et al. 2012).

The present work focuses on BS, a shrinking disorder affecting some *Vitis vinifera* cultivars of highly economic relevance, including Cabernet Sauvignon (Krasnow et al. 2009b; Bondada and Keller 2012), Sauvignon blanc (Raifer et al. 2014) and Blauer Zweigelt (Knoll et al. 2010; Griesser et al. 2012). The symptoms associated with BS have been well described as loss of turgor, high acidity, low pH, low anthocyanin and sugar contents as well as cell death in the berry mesocarp and the rachis (Krasnow et al. 2009b; Hall et al. 2011; Bondada and Keller 2012; Griesser et al. 2012; Bondada 2014). However, the actual causes of BS remain unknown, and the timescale of BS symptom induction is under discussion. The current knowledge suggests no effects on berry development until veraison as seeds are fully developed and able to germinate (Hall et al. 2011; Bondada and Keller 2012).

Grape quality strongly relies on contents of sugars and secondary metabolites. Grape berry cells accumulate hexoses, especially glucose and fructose, in their vacuoles with concentrations up to 1 M when fully ripen (Fontes et al. 2010). This high sugar content accumulates after veraison during the ripening process, when sugars are unloaded from phloem through an apoplastic process (Coombe 1992; Zhang et al. 2006; Kuhn et al. 2014). Veraison marks the shift from symplastic to apoplastic phloem unloading in grapevine berries (Zhang et al. 2006). The combined action of cell wall invertases (CWI) and hexose transporters (HT) retrieves sucrose from the apoplast into berry mesocarp cells (Hayes et al. 2007). Further transport into vacuoles occurs either by sucrose transporters or after cleavage through cytosolic invertases by tonoplast monosaccharide transporters (TMT) (Kuhn et al. 2014; Lecourieux et al. 2014). Polyphenols are among the compounds contributing to sensorial and visual attributes of wines. Their general functions in plants mainly lie in seed dispersion (Harborne 1965), and plant defense towards abiotic (like UV-B light) and biotic stresses (Carbonell-Bejerano et al. 2014). Anthocyanins give the color in red skinned grape varieties and are produced by the phenylpropanoid and flavonoid pathways with phenylalanine as precursor (Sparvoli et al. 1994). Color intensity strongly depends on the concentration and composition of anthocyanins, whose biosynthetic pathways and external factors influencing these routes are well characterized (Kliewer 1970; Dokoozlian and Kliewer 1996; Keller and Hrazdina 1998; Bogs et al. 2007; Mori et al. 2007). The correlation between sugar accumulation and anthocyanin biosynthesis has been observed in vivo and in vitro, showing differences in anthocyanin production depending on type and concentrations of hexoses and sucrose (Afoufa-Bastien et al. 2010; Acevedo et al. 2012; Dai et al. 2014). Abscisic acid (ABA) has been identified as a triggering factor for anthocyanin biosynthesis (Castellarin et al. 2011), although sucrose has been suggested to induce anthocyanin biosynthesis without the need of ABA (Dai et al. 2014).

Main BS symptoms are low anthocyanin contents and blockage of sugar accumulation in berries. The physiological background leading to these observed symptoms and the knowledge of the molecular mechanisms behind BS induction and BS symptoms are very limited. Therefore, the objectives of the present study are to analyze key functions in polyphenol metabolism and sink establishment in pre-symptomatic as well as symptomatic BS berries in comparison to healthy berries. Thereby we aim (1) to analyze the polyphenol production in BS symptomatic grapes and the expression of polyphenol biosynthesis related genes in both pre-symptomatic and symptomatic BS berries, and (2) to monitor the phloem unloading process in pre-symptomatic and symptomatic BS berries analytically by HPAEC-PAD and the expression of key genes responsible for sugar cleavage, transport and sink activity.

## Materials and methods

### Plant material

Plant material used in this and a previous study (Griesser et al. 2017) was obtained from distal parts of grape clusters from a commercial vineyard in lower Austria (Antlasberg, Mailberg GPS coordinates 48.6667, 16.1833), which has a history of high BS incidence according to the owner. The vineyard was planted in 1974 with the *Vitis vinifera* L. cultivar Zweigelt (syn. Blauer Zweigelt) grafted on Kober 5BB (*V. riparia* × *V. berlandieri*) with vertical shoot with bilateral canes as trellising system and individual vine space of 4.2 m^2^ and a yield expectation of 9000 kg per hectare without any cluster thinning. The details concerning soil composition and nutrients availability are described elsewhere (Griesser et al. 2017). In total, 300 grape clusters were randomly labeled within the vineyard 2 weeks after anthesis (16.06.2011). Starting with BBCH79 (majority of berries touching), soluble solids (Digital refractometer, Atago PAL-1, Tokyo, Japan) were determined on a weekly basis on single berries of all labeled grape clusters. Ten individual berries (pedicel included) were collected from labeled clusters every week, and they were immediately frozen in liquid nitrogen. Berries were collected at six sampling time points [from BBCH79 till BBCH89 (berries ripe for harvesting)] for further analyses: 22.07. (42 days after anthesis, DAA), 29.07. (49 DAA), 4.08. (55 DAA), 11.08. (62 DAA), 17.08. (68 DAA) and 24.08. (75 DAA) (Fig. S1 and Fig. S2). They were sampled from each cluster only once to allow an adequate a posteriori categorization of labeled grape clusters into healthy or BS-affected. Such categorization occurred on the 09.09.2011 by visual evaluation and by soluble solids measurement. In total, 37% of labeled grape clusters developed BS symptoms. No differences were observed between proximal and distal clusters, as previously evidenced (Knoll et al. 2010). Frozen berries were selected according to their a posteriori categorization, and used for further analyses with four biological replicates per sample category and sampling time point (total 48 samples). Each of the samples used for analyses was a mix of two probes collected in the vineyard. Seeds were removed and plant material was homogenized with a ball mill (Retsch MM400, Haan, Germany) under cold conditions to prevent thawing.

### Measurement of total anthocyanin content

Total anthocyanin content was measured following the pH differential method (Wrolstad et al. 2005; Fang et al. 2011; Griesser et al. 2012). Four biological replicates were used and results were corrected to the dry weight (DW).

### Polyphenol analysis

Anthocyanins and other selected polyphenols were extracted and analyzed using a validated liquid chromatography–tandem mass spectrometry (LC–MS/MS) method (Schoedl et al. 2011). Analyses were performed with grape berry samples collected on the 24.08.2011 (75 DAA). 100 mg of homogenized plant material was extracted twice each for 10 min in 1 mL of extraction solvent [0.02% hydrochloric acid (v/v) in 80% aqueous MeOH (v/v)] using ultrasonication in ice cold water. The combined extracts were diluted 1:1 with 0.5% aqueous (v/v) formic acid in water and 5 μL of the diluted extract were injected into the LC–MS/MS system. Detection and quantification was performed using a 4000 QTrap LC–MS/MS system (AB Sciex, Foster City, CA, USA) equipped with a TurboIonSpray electrospray ionization (ESI) source and a 1290 series HPLC system (Agilent, Waldbronn, Germany). Chromatographic separation was obtained at 40 °C on a Gemini RP-18 column, 100 × 2 mm inner diameter, 3 μm particle size (Phenomenex, Torrance, CA, USA) protected with a Gemini 3.0 × 2 mm guard column (Phenomenex) using gradient elution. The mobile phase consisted of (A) 0.5% formic acid in H_2_O and (B) 0.5% formic acid in MeOH. The flow rate was 400 µL min^−1^ and the total run time 22 min. Selected reaction monitoring (SRM) mode in negative polarity was performed with the following settings: source temperature, 550 °C; curtain gas, 10 psi (69 kPa of maximum 99.5% nitrogen); ion source gas 1 (source heating gas), 50 psi (345 kPa of nitrogen); ion source gas 2 (drying gas), 50 psi (345 kPa of nitrogen); ion spray voltage, −4000 V. External calibration was applied for quantification (Schoedl et al. 2011). To putatively identify conjugated anthocyanins in a second analysis step, SRM transitions (De la Cruz et al. 2012; Sapozhnikova 2014) were included into the method and acquainted using the positive ionization mode.

### Sugar analysis

Extraction and analysis of carbohydrates were performed as previously described (Guignard et al. 2005). Briefly, 100 mg of powdered grape material was mixed with 1 mL of ethanol 80%. This mixture was homogenized using a vortex for 30 s and shaken for 30 min at 4 °C. After centrifugation at 10,000*g* for 10 min at 4 °C, the supernatant was collected. An additional extraction was done on the pellet using the same extraction solvent (0.5 mL). The supernatants were pooled and evaporated to dryness in a SpeedVac concentrator (Heto, Thermo Electron Corporation, Waltham, MA, USA). Carbohydrates were re-suspended in 1 mL of water and the suspension was filtered through a 0.45 µm Acrodisc PVDF syringe filter (Sigma Aldrich, St. Louis, MO, USA) prior to analysis using high performance anion exchange chromatography coupled with pulsed amperometric detection (HPAEC-PAD).

### RNA extraction and qPCR analyses

Total RNA was extracted from frozen berries according to a modified protocol (Reid et al. 2006) and qPCRs were performed as described in Griesser et al. (2017). The following chemicals were used: Amplification Grade DNase 1 (Sigma Aldrich) for DNA digestion, GoScript Reverse Transcription System (Promega, Madison, WI, USA) for cDNA synthesis, 2X KAPA SYBR FAST qPCR Universal (Peqlab, Erlangen, Germany) for qPCRs. Cycling conditions were as follows: activation 4 min at 95 °C, 40 cycles for 8 s at 95 °C, 20 s at 60 °C, 30 s at 72 °C and 5 s at 75 °C with fluorescence measurement. Most stable reference genes were tested according to Normfinder calculation (Andersen et al. 2004). Among the tested reference genes (actin (VIT_04s0044g00580), ef1 (VIT_06s0004g03220), gadph (VIT_17s0000g10430) and ubiquitin (VIT_16s0098g01190)), actin and ubiquitin gave the best combination for our qPCR analyses. Genes related to the anthocyanin pathway and sugar metabolism and transporter were tested with qPCR (Table S1).

### Enzyme activity

Vacuolar invertase (VI), cell wall invertase (CWI) and cytosolic invertase (CI) assays were performed with 40 mg of whole berry fresh weight (FW) [adapted from (Gibon et al. 2004)]. Soluble proteins (VI and CI) were obtained after incubation in 400 µL of extraction buffer (50 mM Hepes, 0.25% BSA, 10 mM MgCl_2_, 1 mM EDTA, 1 mM EGTA, 1 mM E-aminocaproic acid, 1 mM benzamidine, 25 mM sorbitol, 2 mM leupeptin, 50 mM phenylmethylsulfonyl fluoride (PMSF), 10 mM DTT, 1% triton, 20% glycerol, 1% polyvinylpyrrolidone (PVP), 1% polyvinylpyrrolidone (PVPP) for 10 min on ice and centrifugation at 4 °C with 10,000*g* for 10 min. From the resulting pellet, insoluble proteins were extracted in 400 µL NaCl high salt buffer (1 M NaCl, 50 mM Hepes, 0.25% BSA, 10 mM MgCl_2_, 1 mM EDTA, 1 mM EGTA, 1 mM E-aminocaproic acid, 1 mM benzamidine, 25 mM sorbitol, 2 mM leupeptin, 50 mM PMSF, 10 mM DTT, 1% triton, 20% glycerol, 1% PVP, 1% PVPP for CWI reaction). VI and CWI quantification occurred in a two-step assay with sodium acetate/KOH pH 4.5. Reactions (5 µL extract, reaction volume 20 µL) started by adding of 20 mM sucrose for 20 min followed by an inactivation step at 95 °C. Glucose and fructose formations were monitored in a second step at pH 7 after adding 80 µL detection buffer (0.2 M Hepes pH 7, 1 U mL^−1^ glucose-6-phosphate-dehydrogenase (G6PDH), 1 U mL^−1^ hexokinase (HXK), 1 mM ATP and 1 mM NAD). NADH synthesis was quantified at 340 nm (Microplate reader FLUOstar Omega, BMG Labtech, Offenburg, Germany). CI activity tests were run in a one-step assay as invertase reaction and detection were performed simultaneously at pH 7 (50 mM Hepes pH 7, 20 mM sucrose in the samples, 1 U mL^−1^ G6PDH, 1 U mL^−1^ HXK, 1 mM ATP and 1 mM NAD). Standards calibration curves were included and calculations were performed with the software Optima MARS Data Analysis (BMG Labtech, Offenburg, Germany) by the slope linear regression function. Results are expressed as nmol/min.

### Statistical analysis

qPCR expression analyses were calculated as normalized relative quantities (NRQ) with the R package easyqPCR (Le Pape 2012). All statistical comparisons were conducted using IBM SPSS Statistics 21. Normal distribution of data sets was tested using the Shapiro–Wilk test. Outliers were determined according to Grubb interquartile rules for sugars and qPCR analyses. Significant differences were tested by comparison of healthy and BS-affected berries with Student’s *t* test (*P* < 0.05) if normal distribution was ensured, otherwise non-parametric Mann–Whitney *U* tests were conducted.

## Results

### Effects of BS induction on polyphenol biosynthesis

**Fig. 1:**
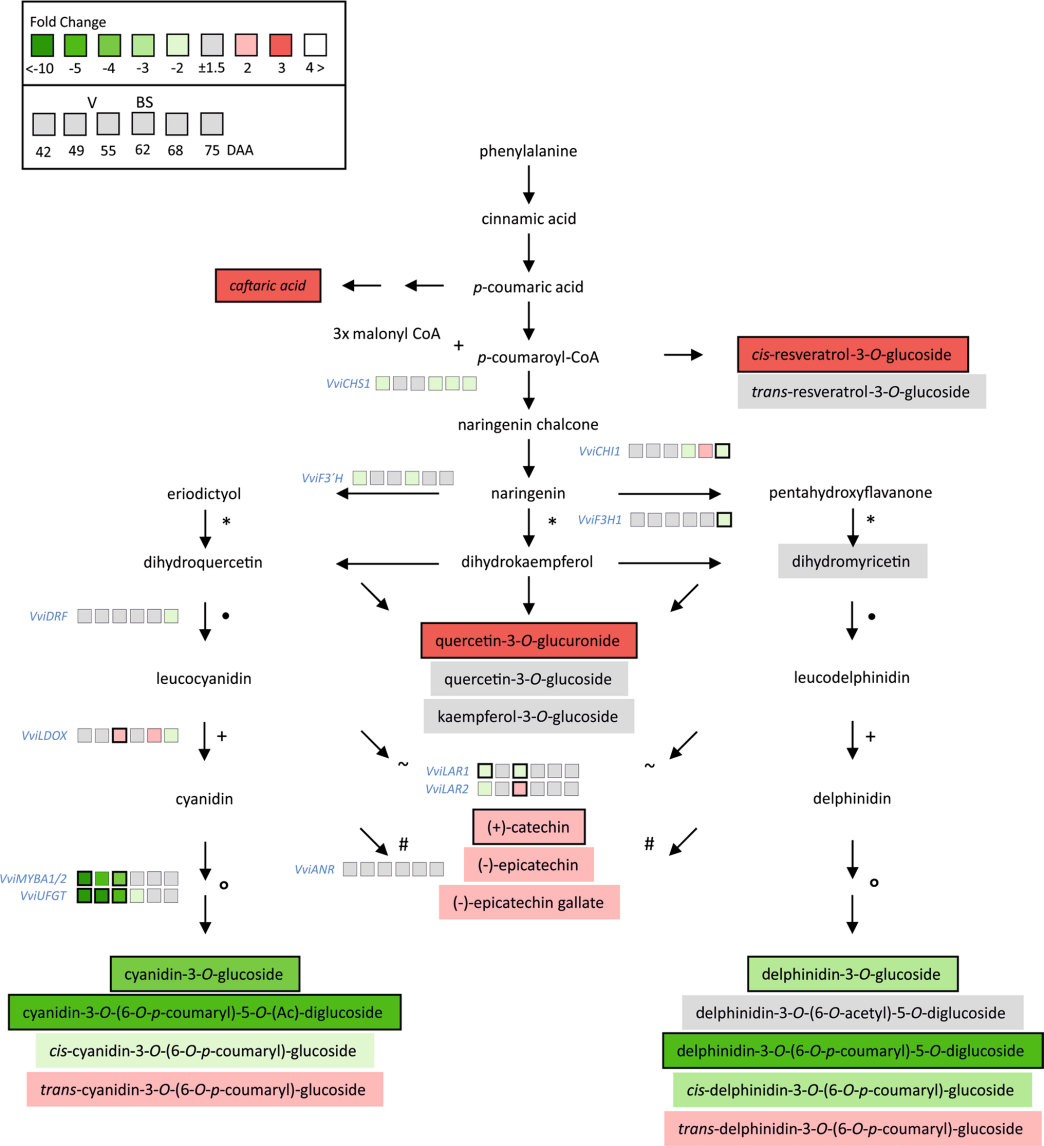
The simplified scheme of the phenylpropanoid and flavonoid pathways combines results obtained for polyphenols with LC–MS/MS and qPCR with selected genes along the biosynthesis pathway. Data are calculated as fold changes (BS in relation to H). Plant material collected on the 24.08.2011 (75 DAA) was used to determine polyphenols in grapevine berries (*n* = 4 each, *P* < 0.05). Compounds analyzed are named in square boxes. Timescale expression analyses with qPCR of selected genes (blue) were performed 42, 49, 55, 62, 68 and 75 DAA, and results are presented as six boxes near the gene name. The start of ripening (veraison, V) was at 55 DAA and the observation of first obvious symptoms (BS) was at 62 DAA. Green and red boxes indicate down- or up-regulation of the genes and abundance of metabolites, respectively. Bold frames identify significant differences (*n* = 4 each, *P* < 0.05) between healthy and BS samples. All data (compounds analyzed and expression as NRQ) are shown in Tables S2 and S3

A reduction in total anthocyanins and soluble solids was observed in BS affected berries starting from 75 DAA (Fig. S3b). A highly selective and sensitive LC–MS/MS method was applied to a subset of samples (75 DAA) to further investigate polyphenol metabolism in BS grape berries. Additionally, we analyzed the expression level of key phenylpropanoid and flavonoid biosynthetic genes in pre-symptomatic and symptomatic BS berries. Both results are schematically shown in Fig. 1. Among the 32 tested polyphenols, some were found to be under the detection limit (Table S2), including anthocyanidins [aglycons of the anthocyanins delphinidin (DEL) and cyanidin (CYA)]. Therefore, further analyses were conducted to analyze the conjugated forms of these analytes. Nine CYA and DEL metabolites were putatively identified based on mass spectra and chromatographic retention behavior, as suggested in similar studies (Acevedo et al. 2012). For the comparative analysis between healthy and BS samples, the respective integrated peak areas were compared. This detailed analysis revealed that delphinidin-glucosides as well as one of its conjugate (putatively identified as DEL-3-*O*-(6-*O*-p-coumaroyl)-5-*O*-diglucoside,) and cyanidin-glucoside and one of its derivatives (CYA-3-*O*-(6-*O*-p-coumaroyl)-5-*O*-(Ac)-diglucoside) were less abundant in BS than in healthy samples (Fig. 1). On the contrary, strong increases in caftaric acid, *trans*-resveratrol-3-*O*-glucoside, quercetin-3-*O*-glucuronide and (+)-catechin were observed in BS samples compared to the healthy ones, indicating a differential distribution of assimilates towards earlier branch points within the phenylpropanoid pathway (Fig. 1). Expression analyses of thirteen key genes of the phenylpropanoid and flavonoid pathways were performed on a timescale with pre-symptomatic and symptomatic (between 42 and 75 DAA) BS berries. In general, gene expression was very similar between healthy and BS berries. Differences ranged, with some exceptions between +2 and −2 fold change, and only some comparisons were found to be significantly different [e.g., *VviCHI1* and *VviF3H1* with a lower expression at the last sampling date (75 DAA)]. Expression data as NRQs are shown in Table S3. We observed a significant difference for *VviUFGT* and *VviMYBA1/2* expression levels before veraison, with a lower expression (five- to tenfold) in pre-symptomatic BS berries compared to the healthy ones. With ongoing development, the expression of these genes equalizes between healthy and BS berries. Additionally, transcripts of two genes involved in tannin biosynthesis (*VviLDOX* and *VviLAR2*) were found to be increased at veraison in BS berries.

### Sink activity in BS affected grapes

**Fig. 2:**
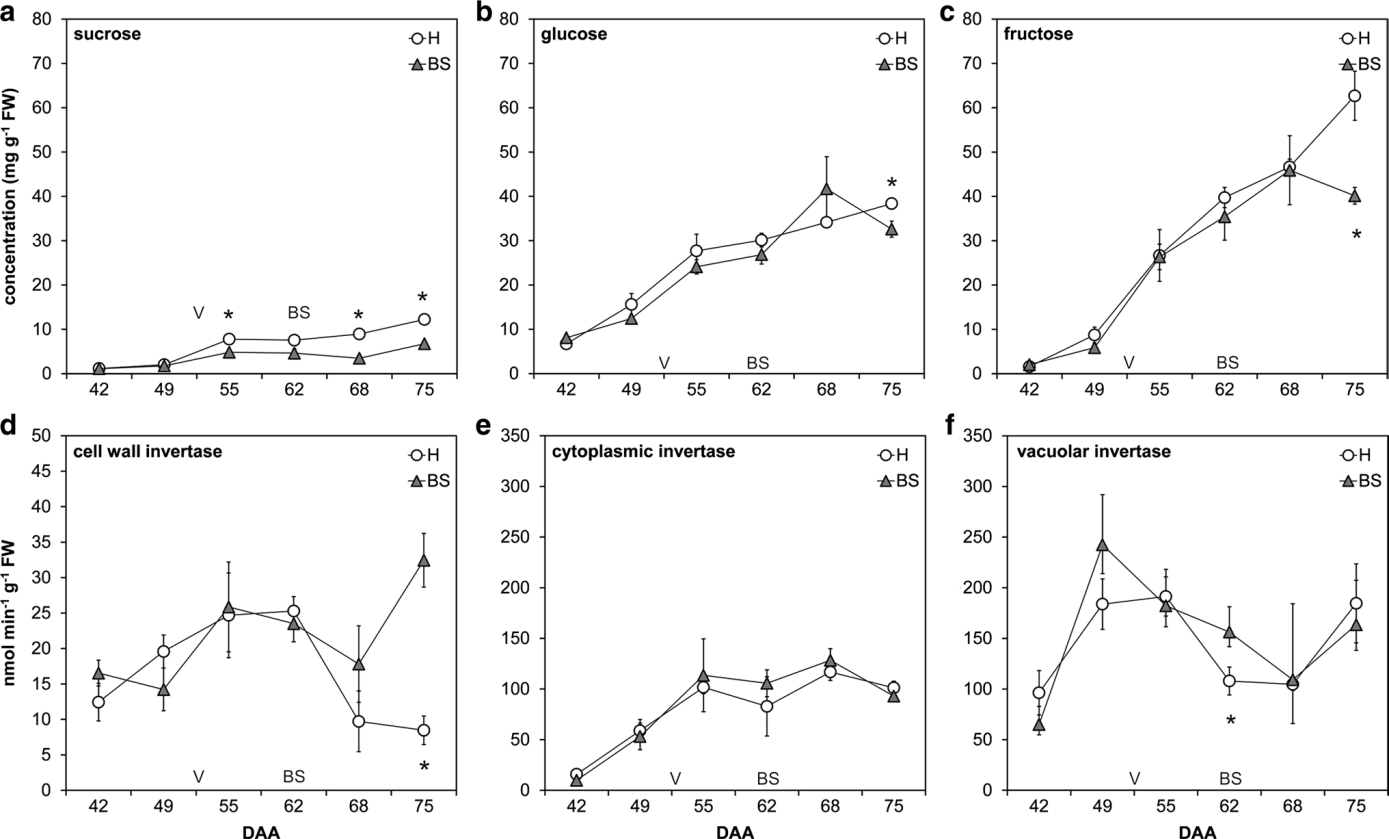
Sucrose (**a**), glucose (**b**) and fructose (**c**) content in healthy (H) and berry shrivel (BS) grape clusters at different developmental stages (days after anthesis, DAA) is shown as well as the timescale of enzymatic activity of cell wall invertase (CWI) (**d**), cytosolic invertase (CI) (**e**) and vacuolar invertase (VI) (**f**). Veraison as starting point for ripening is indicated (V) and the first BS symptoms were observed 62 DAA. Data presented are mean values on fresh weight basis ± standard error (*n* = 4 each) and statistical significant differences are indicated with an asterisk (*P* < 0.05)

Berries become strong sinks and accumulate sugars and nutrients at ripening. Berries affected with BS stop the increase of soluble solids at about 11–13°Brix short after veraison, whereas berries from healthy grape clusters continue to values up to 20°Brix (Fig. S1). Reduced Brix levels were obtained at 75 DAA, while the trend could already be observed 55 DAA at veraison. Detailed analyses of sugars were performed throughout the growing season (Fig. 2a–c). Healthy berries accumulated sugars throughout the growing season, whereas sugars content (especially sucrose) was lower in BS berries, starting at 55 DAA. Regarding hexose, no significant differences were detected until 75 DAA.

**Fig. 3:**
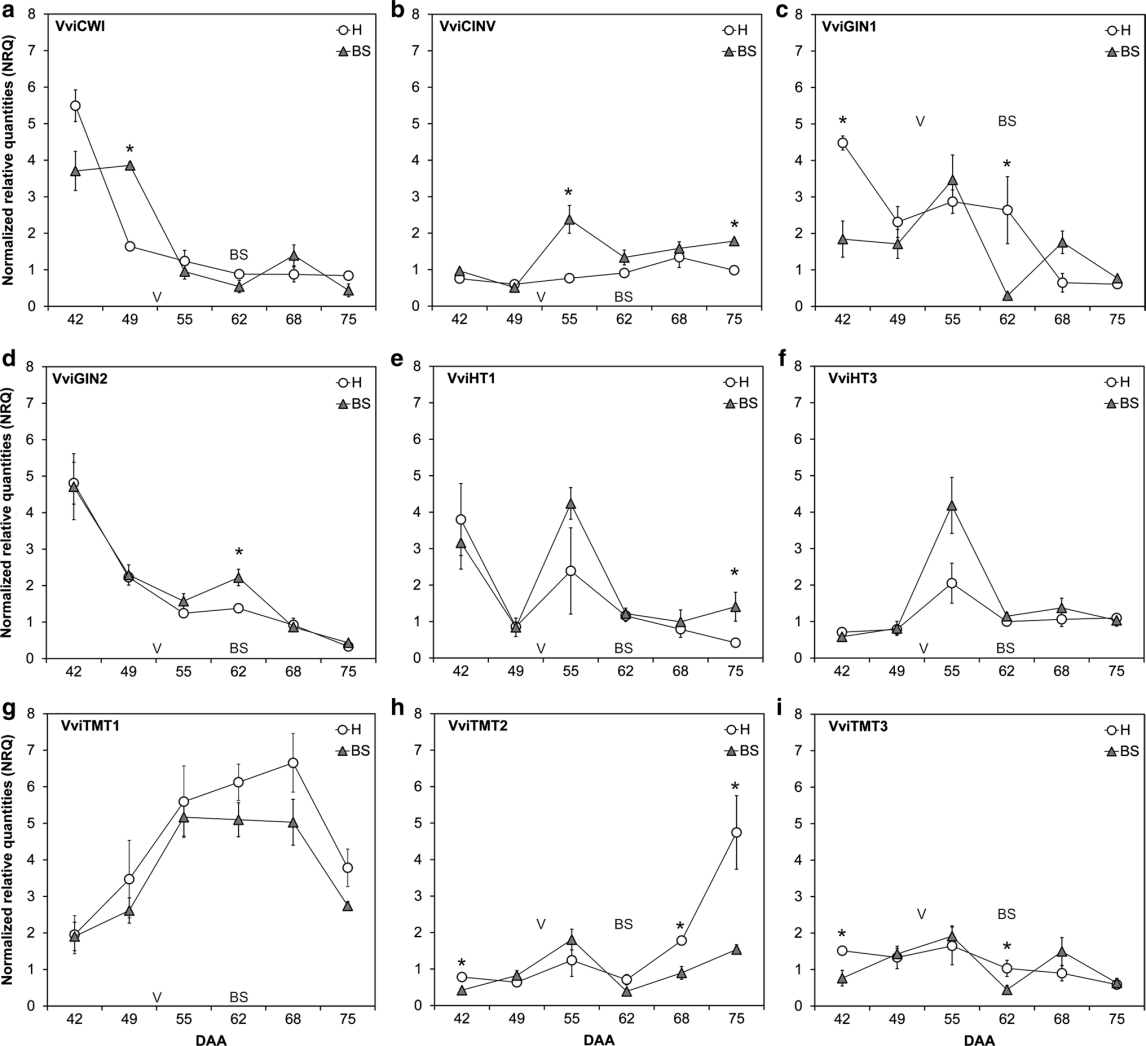
Relative expression of *VviCWI* (**a**), *VviCINV* (**b**), *VviGIN1* (**c**), *VviGIN2* (**d**), *VviHT1* (**e**), *VviHT3* (**f**), *VviTMT1* (**g**), *VviTMT2* (**h**) and *VviTMT3* (**i**) in berries of healthy (H) and berry shrivel symptomatic (BS) grapes at different developmental stages (days after anthesis, DAA) are shown as NRQs. Veraison as starting point for ripening is indicated (V) and the observation of first obvious symptoms (BS) was 62 DAA. Data shown are normalized relative quantities, representing mean values ± standard error and statistical significant differences are indicated with an asterisk (*n* = 4 each, *P* < 0.05)

The quantification of enzymes of the carbohydrate metabolism did not provide conclusive results in terms of sink activity (Fig. 2d–f). VI was found to be higher in BS than in healthy berries around veraison and when the first BS-related symptoms are observable, whereas no differences between healthy and BS berries were detected during the ripening process. The amount of CWI is much higher in BS berries than in the healthy ones 68 DAA, when BS symptoms are already visible. No differences in cytosolic invertase (CI) between healthy and BS grapes were obtained at the analyzed time points. As a whole, gene expression results did not provide a sound overview either, meaning similar responses of several genes during BS induction involved in sink establishment and activity. Nevertheless, interesting individual results were obtained (Fig. 3a–i). The expression levels of *VviCWI* and *VviCINV1* genes are higher in BS berries around veraison (49 and 55 DAA), whereas *VviGIN1* transcript is strongly reduced before veraison (42 DAA). Furthermore, the expression of *VviGIN2* is increased 62 DAA. Regarding sugar metabolism, *VviTMT2* and *VviTMT3* are of special interest, as they are responsible for hexose transport into vacuoles and their expression is repressed before veraison (42 DAA). During the ripening process (68 and 75 DAA) *VviTMT2* expression was found to be strongly reduced in BS berries if compared to healthy berries. Expression data as NRQs are shown in Table S3.

## Discussion

The main symptoms of the physiological ripening disorder BS are lower soluble solids and total anthocyanin contents shortly after the onset of berry ripening. Despite the importance of sugars and anthocyanins for grape berry quality, no details on both processes during BS induction and symptom development have been reported so far. Herein we present results of an integrative approach to investigate and characterize the sugar transport and metabolism and the biosynthesis of polyphenols in symptomatic and pre-symptomatic grape berries.

Gene expression analyses revealed lower transcript levels of the anthocyanin biosynthesis genes *VviUFGT*, *VviMYBPA1* and *VviMYBA1/2* in BS berries before and around veraison. These three genes are responsible for the final step of anthocyanin biosynthesis in grape skins, and *VviMYBA1/2* was proposed as an early molecular marker for color development (Castellarin et al. 2011). Therefore, the start of ripening in grapes developing BS symptoms seems to be disturbed with respect to anthocyanin biosynthesis. Analytical determination of polyphenols (and especially anthocyanins) confirmed expression analyses. Reduced amounts of delphinidin glucoside and cyanidin glucoside conjugate forms were detected in BS berries, while caftaric acid, some flavonols and flavan-3-ols and other non-glycosylated phenylpropanoid conjugates were present in slightly higher amounts. Glycosylated molecules usually have higher energetic costs for their biosynthesis, which could be a limiting factor in the case of BS berries (Teixeira et al. 2013). The main metabolic precursors for polyphenols are sucrose and phenylalanine. Whereas similar content in BS and healthy berries have been reported for the amino acid phenylalanine (Griesser et al. 2012), we found a reduced content of sucrose in BS berries. Sucrose and hexoses have an important role as signaling molecules that trigger the expression of genes involved in anthocyanin biosynthesis (Hiratsuka et al. 2001; Castellarin et al. 2011). The necessity of a given concentration of sugar for triggering anthocyanin synthesis has been described in in vitro culture berries (Dai et al. 2014). Apart from sugars, the onset of ripening in grape berries is coordinated by phytohormones, and the contribution of ABA (Davies et al. 1997), auxins (Bottcher et al. 2010), brassinosteroids (Symons et al. 2006) and ethylene (Sun et al. 2010) is already known. At veraison, the content of auxins are reduced, whereas ABA increases dramatically starting the ripening process, driving to berry softening and anthocyanin biosynthesis in the skin of red varieties (Davies and Robinson 2000). A delayed reduction of auxin biosynthesis might result in no differences in total anthocyanins content in berries at the end of the ripening season (Bottcher et al. 2012) and it is therefore unlikely to be the cause of the BS symptoms observed. Indeed, auxin content in BS berries is not increased as compared to healthy berries at the onset of ripening. In case of active ABA, the differences were minor, whereas ABA-glucose ester (ABA-GE) contents were much higher in already symptomatic BS berries (unpublished data). In general, ABA can accelerate ripening, while the absence of ABA delays it (Mori et al. 2007; Wheeler et al. 2009). A slightly reduced content of active ABA was detected in pre-symptomatic BS berries, but the contribution to BS induction and symptoms development will have to be evaluated in future experiments. In addition, the role of ABA-GE as the transport form of ABA in BS berries needs further consideration in terms of abiotic stress response. Additionally, the process of cell turgor decrease and fruit softening has to be considered as ripening starts (Castellarin et al. 2011). The expression of *VviMYBA1/2* and *VviUFGT* has been shown to be closely linked to osmotic potential, which highly depends on sugar accumulation and seems to be important in the regulation of color development and probably other ripening processes (Wada et al. 2009; Castellarin et al. 2011). Unfortunately, osmolality was not determined in this work, but this measurement could provide additional information in terms of cell compartmentation. Additionally, timescale analyses of polyphenols would be interesting, as samples used in this study did already show BS symptoms.

Sugar accumulation in grape berries starts at veraison, which is associated with a shift from a predominately symplastic towards an apoplastic phloem unloading. Invertases and hexose transporters play important roles in this process of sink establishment (Zhang et al. 2006). Sugar accumulation is hindered in BS berries, as reflected in the soluble solids and sugar analyses performed in this study. The differences in the content of hexoses and sucrose in BS berries compared to berries without symptoms were much lower as we could have expected from soluble solid measurements. In this regard, berry shrinking could have contributed to the concentration of sugars in BS berries once obvious symptoms became visible. The slightly reduced hexose and sucrose contents in BS berries before veraison could be more important for BS induction. Expression analyses in pre-symptomatic BS berries revealed a reduction of *VviGIN1*, *VviTMT2* and *VviTMT3* transcripts, whereas no difference in the expression level of *VviHT1* was detected. In parallel, the expression of *VviCWI* is first slightly lower and 1 week later increased in BS berries before veraison. CWI enzyme is increased in BS berries after BS symptoms are visible, but no differences in enzyme quantities were observed before and around veraison. Cell wall invertase activity before and at veraison is an important step to establish grape berries as sink tissue (Davies and Robinson 1996; Zhang et al. 2006). Combining these data, the phloem unloading of sucrose seems to be well-established in BS berries, whereas the transport into vacuoles could be affected. It is also possible that on a relative scale more sucrose is entering the cytosol of mesocarp cells of BS berries before veraison, as the expression of *VviCINV* and *VviGIN1* is higher in BS berries compared to the healthy ones. Cells must sense the osmotic potential for berry softening, relax the cell walls and control cell turgor for cell expansion (Castellarin et al. 2016). Disturbance in cell compartmentation would highly affect this process of cell growth, and as a consequence, the phloem unloading process and ripening itself. Phytohormones could influence the process, as ethylene and gibberellins influence the expression of sugar transporters and enzymes in grape berries (Chervin et al. 2006; Cheng et al. 2015). In pre-symptomatic BS berries, GA19 is reduced whereas ACC as ethylene precursor is strongly increased (unpublished data). Therefore, direct conclusions are difficult to draw, as different processes seem to be affected.

Other causes for reduced sugar and anthocyanin levels in grapes are plant virus infections. The grapevine leaf-roll-associated virus-2 (GLRa V-3) influences sugar and flavonoid metabolism in leaves and berries. Previous works indicate that fructose and glucose accumulation and the expression of *VviHT1* and *VviMSA* genes can be reduced during the ripening process of virus-infected vines. Same effect was determined for the anthocyanin content and the expression levels of *VviUFGT* and *VviMYBA1* genes (Vega et al. 2011). Biotic causes for BS have not been identified, but due to similarity of symptoms in terms of sugar accumulation and anthocyanin biosynthesis, a deep survey for viruses and endophytes should be conducted.

## Conclusion

The ripening disorder BS affects anthocyanin biosynthesis and the expression of some sugar metabolism genes. As specific induction of BS is not possible, the sampling strategy in vineyards to collect symptomatic and pre-symptomatic grapes is a crucial part for its in-depth analysis. With the current knowledge, it is very difficult to distinguish between primary effects and follow-up symptoms of the BS induction process. One of the key questions to identify the causes of BS is the time point of BS induction. Our data indicate metabolic changes before veraison, including different levels of *VviGIN1*, *VviTMT2*, *VviTMT3*, *VviMYBA1/2* and *VviUFGT* transcripts. The slightly reduced glucose and fructose contents observed in BS affected grapes before veraison could be a consequence of the observed different expression levels for invertases and sugar transporters genes. The low hexose reserves may have delayed the anthocyanin biosynthesis, as it is reflected in a reduced expression of the transcription factor and the biosynthesis gene. The role of phytohormones and other enzymes involved in the berry softening process needs to be considered to complete the picture. BS induction is a complex process and broad scale data analyses (RNASeq, metabolites) are necessary to distinguish between causing events and follow-up processes.

### *Author contribution statement*

MG, SCM and AF conceived and designed the experimental setup, performed data analyses and wrote the paper. SCM performed qPCR of sugar metabolism genes and carbohydrate enzymatic assays. ME performed qPCRs of polyphenol biosynthesis genes. BW analyzed polyphenols and annotated conjugated anthocyanin conjugates. CA analyzed sugars. RS supervised LC–MS measurements and contributed in writing the paper. All authors discussed the results and revised the manuscript.

## Electronic supplementary material

Below is the link to the electronic supplementary material.

Supplementary material 1 (DOCX 1050 kb)

## Abbreviations

BS: Berry shrivel
CWI: Cell wall invertase
DAA: Days after anthesis
V: Veraison

## References

[CR1] Acevedo CA, Creixell W, Pavez-Barra C, Sánchez E, Albornoz F, Young ME (2012). Modeling volatile organic compounds released by bovine fresh meat using an integration of solid phase microextraction and databases. Food Bioprocess Tech.

[CR2] Afoufa-Bastien D, Medici A, Jeauffre J, Coutos-Thevenot P, Lemoine R, Atanassova R, Laloi M (2010). The *Vitis vinifera* sugar transporter gene family: phylogenetic overview and macroarray expression profiling. BMC Plant Biol.

[CR3] Andersen CL, Jensen JL, Orntoft TF (2004). Normalization of real-time quantitative reverse transcription-PCR data: a model-based variance estimation approach to identify genes suited for normalization, applied to bladder and colon cancer data sets. Cancer Res.

[CR4] Bogs J, Jaffé FW, Takos AM, Walker AR, Robinson SP (2007). The grapevine transcription factor *VvMYBPA1* regulates proanthocyanidin synthesis during fruit development. Plant Physiol.

[CR5] Bondada B (2014). Structural and compositional characterization of suppression of uniform ripening in grapevine: a paradoxical ripening disorder of grape berries with no known causative clues. J Am Soc Hortic Sci.

[CR6] Bondada B, Keller M (2012). Morphoanatomical symptomatology and osmotic behavior of grape berry shrivel. J Am Soc Hortic Sci.

[CR7] Bottcher C, Keyzers RA, Boss PK, Davies C (2010). Sequestration of auxin by the indole-3-acetic acid-amido synthetase GH3-1 in grape berry (*Vitis vinifera* L.) and the proposed role of auxin conjugation during ripening. J Exp Bot.

[CR8] Bottcher C, Boss PK, Davies C (2012). Delaying Riesling grape berry ripening with a synthetic auxin affects malic acid metabolism and sugar accumulation, and alters wine sensory characters. Funct Plant Biol.

[CR9] Carbonell-Bejerano P, Diago M-P, Martínez-Abaigar J, Martínez-Zapater JM, Tardáguila J, Núñez-Olivera E (2014). Solar ultraviolet radiation is necessary to enhance grapevine fruit ripening transcriptional and phenolic responses. BMC Plant Biol.

[CR10] Castellarin SD, Gambetta GA, Wada H, Shackel KA, Matthews MA (2011). Fruit ripening in *Vitis vinifera*: spatiotemporal relationships among turgor, sugar accumulation, and anthocyanin biosynthesis. J Exp Bot.

[CR11] Castellarin SD, Gambetta GA, Wada H, Krasnow MN, Cramer GR, Peterlunger E, Shackel KA, Matthews MA (2016). Characterization of major ripening events during softening in grape: turgor, sugar accumulation, abscisic acid metabolism, colour development, and their relationship with growth. J Exp Bot.

[CR12] Cheng CX, Jiao C, Singer SD, Gao M, Xu XZ, Zhou YM, Li Z, Fei ZJ, Wang YJ, Wang XP (2015). Gibberellin-induced changes in the transcriptome of grapevine (*Vitis labrusca* × *V. vinifera*) cv. Kyoho flowers. BMC Genom.

[CR13] Chervin C, Terrier N, Ageorges A, Ribes F, Kuapunyakoon T (2006). Influence of ethylene on sucrose accumulation in grape berry. Am J Enol Viticult.

[CR14] Conde C, Agasse A, Glissant D, Tavares R, Gerós H, Delrot S (2006). Pathways of glucose regulation of monosaccharide transport in grape cells. Plant Physiol.

[CR15] Coombe BG (1992). Research on development and ripening of the grape berry. Am J Enol Viticult.

[CR16] Dai ZW, Meddar M, Renaud C, Merlin I, Hilbert G, Delrot S, Gomès E (2014). Long-term in vitro culture of grape berries and its application to assess the effects of sugar supply on anthocyanin accumulation. J Exp Bot.

[CR17] Davies C, Robinson SP (1996). Sugar accumulation in grape berries—cloning of two putative vacuolar invertase cDNAs and their expression in grapevine tissues. Plant Physiol.

[CR18] Davies C, Robinson SP (2000). Differential screening indicates a dramatic change in mRNA profiles during grape berry ripening. Cloning and characterization of cDNAs encoding putative cell wall and stress response proteins. Plant Physiol.

[CR19] Davies C, Boss PK, Robinson SP (1997). Treatment of grape berries, a nonclimacteric fruit with a synthetic auxin, retards ripening and alters the expression of developmentally regulated genes. Plant Physiol.

[CR20] De la Cruz AA, Hilbert G, Riviere C, Mengin V, Ollat N, Bordenave L, Decroocq S, Delaunay JC, Delrot S, Merillon JM, Monti JP, Gomes E, Richard T (2012). Anthocyanin identification and composition of wild *Vitis* spp. accessions by using LC–MS and LC–NMR. Anal Chim Acta.

[CR21] Dokoozlian N, Kliewer W (1996). Influence of light on grape berry growth and composition varies during fruit development. J Am Soc Hort Sci.

[CR22] Fang YL, Meng JF, Zhang A, Liu JC, Xu TF, Yu WL, Chen SX, Li H, Zhang ZW, Wang H (2011). Influence of shriveling on berry composition and antioxidant activity of Cabernet Sauvignon grapes from Shanxi vineyards. J Sci Food Agr.

[CR23] Fontes N, Delrot S, Geros H (2010). A method for the isolation of protoplasts from grape berry mesocarp tissue. Recent Pat Biotechnol.

[CR24] Gibon Y, Blaesing OE, Hannemann J, Carillo P, Hohne M, Hendriks JHM, Palacios N, Cross J, Selbig J, Stitt M (2004). A robot-based platform to measure multiple enzyme activities in Arabidopsis using a set of cycling assays: comparison of changes of enzyme activities and transcript levels during diurnal cycles and in prolonged darkness. Plant Cell.

[CR25] Griesser M, Eder R, Besser S, Forneck A (2012). Berry shrivel of grapes in Austria-Aspects of the physiological disorder with cultivar Zweigelt (*Vitis vinifera* L.). Sci Horti-Amst.

[CR26] Griesser M, Martinez SC, Weidinger ML, Kandler W, Forneck A (2017). Challenging the potassium deficiency hypothesis for induction of the ripening disorder berry shrivel in grapevine. Sci Horti-Amst.

[CR27] Guignard C, Jouve L, Bogeat-Triboulot MB, Dreyer E, Hausman JF, Hoffmann L (2005). Analysis of carbohydrates in plants by high-performance anion-exchange chromatography coupled with electrospray mass spectrometry. J Chromatogr A.

[CR28] Hall GE, Bondada BR, Keller M (2011). Loss of rachis cell viability is associated with ripening disorders in grapes. J Exp Bot.

[CR29] Harborne J (1965). Plant polyphenols—XIV: characterization of flavonoid glycosides by acidic and enzymic hydrolyses. Phytochemistry.

[CR30] Hayes MA, Davies C, Dry IB (2007). Isolation, functional characterization, and expression analysis of grapevine (*Vitis vinifera* L.) hexose transporters: differential roles in sink and source tissues. J Exp Bot.

[CR31] Hiratsuka S, Onodera H, Kawai Y, Kubo T, Itoh H, Wada R (2001). ABA and sugar effects on anthocyanin formation in grape berry cultured in vitro. Sci Horti-Amst.

[CR32] Keller M, Hrazdina G (1998). Interaction of nitrogen availability during bloom and light intensity during veraison. II. Effects on anthocyanin and phenolic development during grape ripening. Am J Enol Viticult.

[CR33] Kliewer W (1970). Effect of day temperature and light intensity on coloration of *Vitis vinifera* (L.) grapes. J Am Soc Hortic Sci.

[CR34] Knoll M, Achleitner D, Redl H (2010). Sugar accumulation in ‘Zweigelt’ grapes as affected by “Traubenwelke”. Vitis.

[CR35] Krasnow M, Matthews M, Shackel K (2009). Sugar accumulation disorder may be able to spread from affected tissue to healthy tissue. Am J Enol Viticult.

[CR36] Krasnow M, Weis N, Smith RJ, Benz MJ, Matthews M, Shackel K (2009). Inception, progression, and compositional consequences of a berry shrivel disorder. Am J Enol Viticult.

[CR37] Kuhn N, Guan L, Dai ZW, Wu BH, Lauvergeat V, Gomes E, Li SH, Godoy F, Arce-Johnson P, Delrot S (2014). Berry ripening: recently heard through the grapevine. J Exp Bot.

[CR38] Le Pape S (2012) EasyqpcR: EasyqpcR for easy analysis of real-time PCR data atIRTOMIT-INSERM U1082. IRTOMIT-INSERM U1082, sylvain.le.pape@univ-poitiers.fr., http://irtomit.labo.univ-poitiers.fr/. Accessed 13 Oct 2014

[CR39] Lecourieux F, Kappel C, Lecourieux D, Serrano A, Torres E, Arce-Johnson P, Delrot S (2014). An update on sugar transport and signalling in grapevine. J Exp Bot.

[CR40] Mori K, Goto-Yamamoto N, Kitayama M, Hashizume K (2007). Loss of anthocyanins in red-wine grape under high temperature. J Exp Bot.

[CR41] Raifer B, Haas F, Cassar A (2014). Influence of leaf canopy height on the occurrence of berry shrivel. Vitis.

[CR42] Reid KE, Olsson N, Schlosser J, Peng F, Lund ST (2006). An optimized grapevine RNA isolation procedure and statistical determination of reference genes for real-time RT-PCR during berry development. BMC Plant Biol.

[CR43] Sapozhnikova Y (2014). Development of liquid chromatography-tandem mass spectrometry method for analysis of polyphenolic compounds in liquid samples of grape juice, green tea and coffee. Food Chem.

[CR44] Schoedl K, Forneck A, Sulyok M, Schuhmacher R (2011). Optimization, in-house validation, and application of a liquid chromatography-tandem mass spectrometry (LC–MS/MS)-based method for the quantification of selected polyphenolic compounds in leaves of grapevine (*Vitis vinifera* L.). J Agric Food Chem.

[CR45] Sparvoli F, Martin C, Scienza A, Gavazzi G, Tonelli C (1994). Cloning and molecular analysis of structural genes involved in flavonoid and stilbene biosynthesis in grape (*Vitis vinifera* L.). Plant Mol Biol.

[CR46] Sun LA, Zhang M, Ren J, Qi JX, Zhang GJ, Leng P (2010). Reciprocity between abscisic acid and ethylene at the onset of berry ripening and after harvest. BMC Plant Biol.

[CR47] Symons GM, Davies C, Shavrukov Y, Dry IB, Reid JB, Thomas MR (2006). Grapes on steroids. Brassinosteroids are involved in grape berry ripening. Plant Physiol.

[CR48] Teixeira A, Eiras-Dias J, Castellarin SD, Geros H (2013). Berry phenolics of grapevine under challenging environments. Int J Mol Sci.

[CR49] Vega A, Gutierrez RA, Pena-Neira A, Cramer GR, Arce-Johnson P (2011). Compatible GLRaV-3 viral infections affect berry ripening decreasing sugar accumulation and anthocyanin biosynthesis in *Vitis vinifera*. Plant Mol Biol.

[CR50] Wada H, Matthews MA, Shackel KA (2009). Seasonal pattern of apoplastic solute accumulation and loss of cell turgor during ripening of *Vitis vinifera* fruit under field conditions. J Exp Bot.

[CR51] Wheeler S, Loveys B, Ford C, Davies C (2009). The relationship between the expression of abscisic acid biosynthesis genes, accumulation of abscisic acid and the promotion of *Vitis vinifera* (L.) berry ripening by abscisic acid. Aust J Grape Wine R.

[CR52] Wrolstad RE, Durst RW, Lee J (2005). Tracking color and pigment changes in anthocyanin products. Trends Food Sci Tech.

[CR53] Zhang XY, Wang XL, Wang XF, Xia GH, Pan QH, Fan RC, Wu FQ, Yu XC, Zhang DP (2006). A shift of phloem unloading from symplasmic to apoplasmic pathway is involved in developmental onset of ripening in grape berry. Plant Physiol.

