# Supplementary material for: The ripening disorder berry shrivel affects anthocyanin biosynthesis and sugar metabolism in Zweigelt grape berries
Source: Planta. 2017 Oct 26;247(2):471–81. doi: 10.1007/s00425-017-2795-4 (PMC5778156; doi:10.1007/s00425-017-2795-4)
Supplement: Supplementary file 1 — Supplementary material 1 (DOCX 1050 kb) [file 425_2017_2795_MOESM1_ESM.docx]

**The ripening disorder berry shrivel affects anthocyanin biosynthesis and sugar metabolism in Zweigelt grape berries**

Michaela Griesser^1^*, Sara Crespo Martinez^1^, Markus Eitle^1^, Benedikt Warth^3,4^, Christelle Andre^2^, Rainer Schuhmacher^3^, Astrid Forneck^1^

^1^Division of Viticulture and Pomology, Department of Crop Sciences, University of Natural Resources and Life Sciences, Vienna, Konrad Lorenz Straße 24, 3430 Tulln, Austria

^2^Luxembourg Institute of Science and Technology, Department of Environmental Research and Innovation, avenue des Hauts-Fourneaux, L-4362 Esch/Alzette, Luxembourg

^3^Center for Analytical Chemistry, Department of Agrobiotechnology (IFA-Tulln), University of Natural Resources and Life Sciences, Vienna, Konrad Lorenz Straße 20, 3430 Tulln, Austria

^4^Department of Food Chemistry and Toxicology, University of Vienna, Währingerstraße 38, 1090 Vienna, Austria (current address)

*corresponding author: Michaela Griesser, Division of Viticulture and Pomology, Department of Crop Sciences, University of Natural Resources and Life Sciences, Vienna, Konrad Lorenz Straße 24, 3430 Tulln, Austria; [michaela.griesser@boku.ac.at](mailto:michaela.griesser@boku.ac.at); 0043/1/47654-95814

**Supplementary material**

**
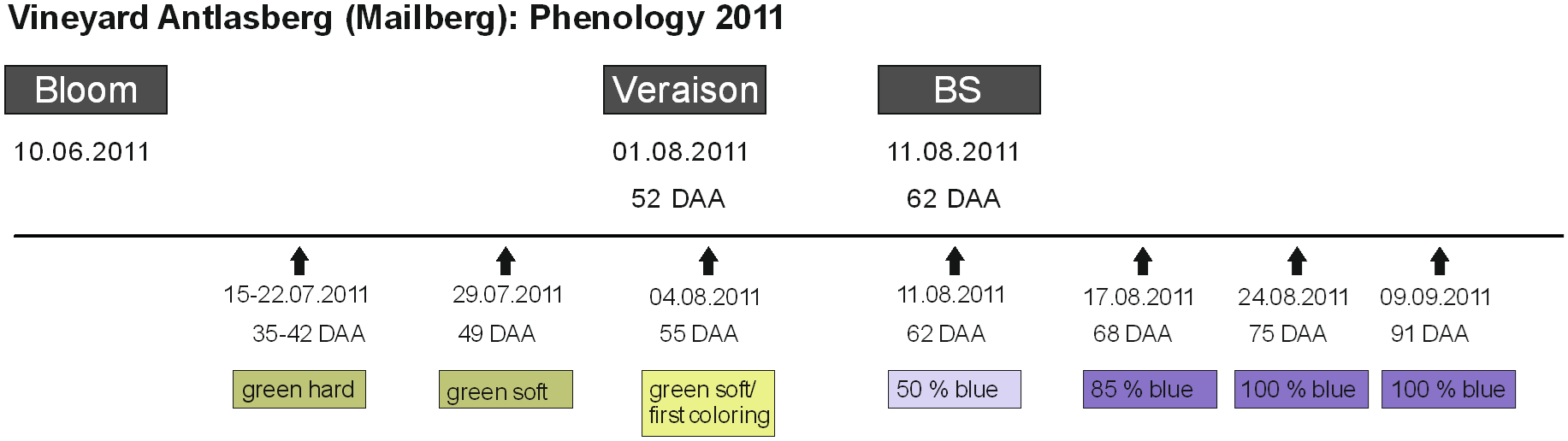
**

**Fig. S1** Phenological development of grapes in the vineyard Anltasberg (Mailberg, Lower Austria) in 2011 and sampling dates. The important phenological stages bloom and veraison occured 2011 in the specific vineyard on the 10.6. and 1.8. respectively. Obvious symptoms of berry shrivel were first observed on the 11.08.2011, when about 50% of berries were colored.


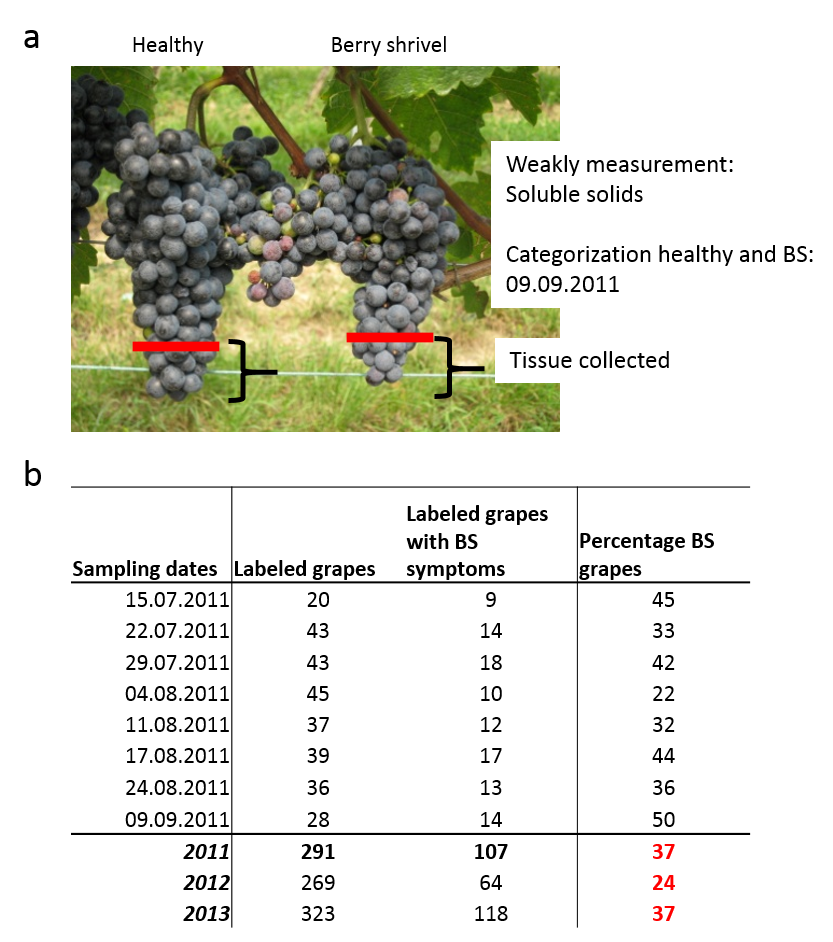


**Fig. S2** (**a**) Sampling strategy and tissue collected of healthy and BS grape clusters. (**b**) Summary of samples collected on each sampling date and the number of BS grapes in 2011, as total numbers and percentages. Additionally the mean number of BS grapes (amounts and percentages) from 2011 till 2013 in the vineyard Anltasberg (Mailberg, Lower Austria) are presented.


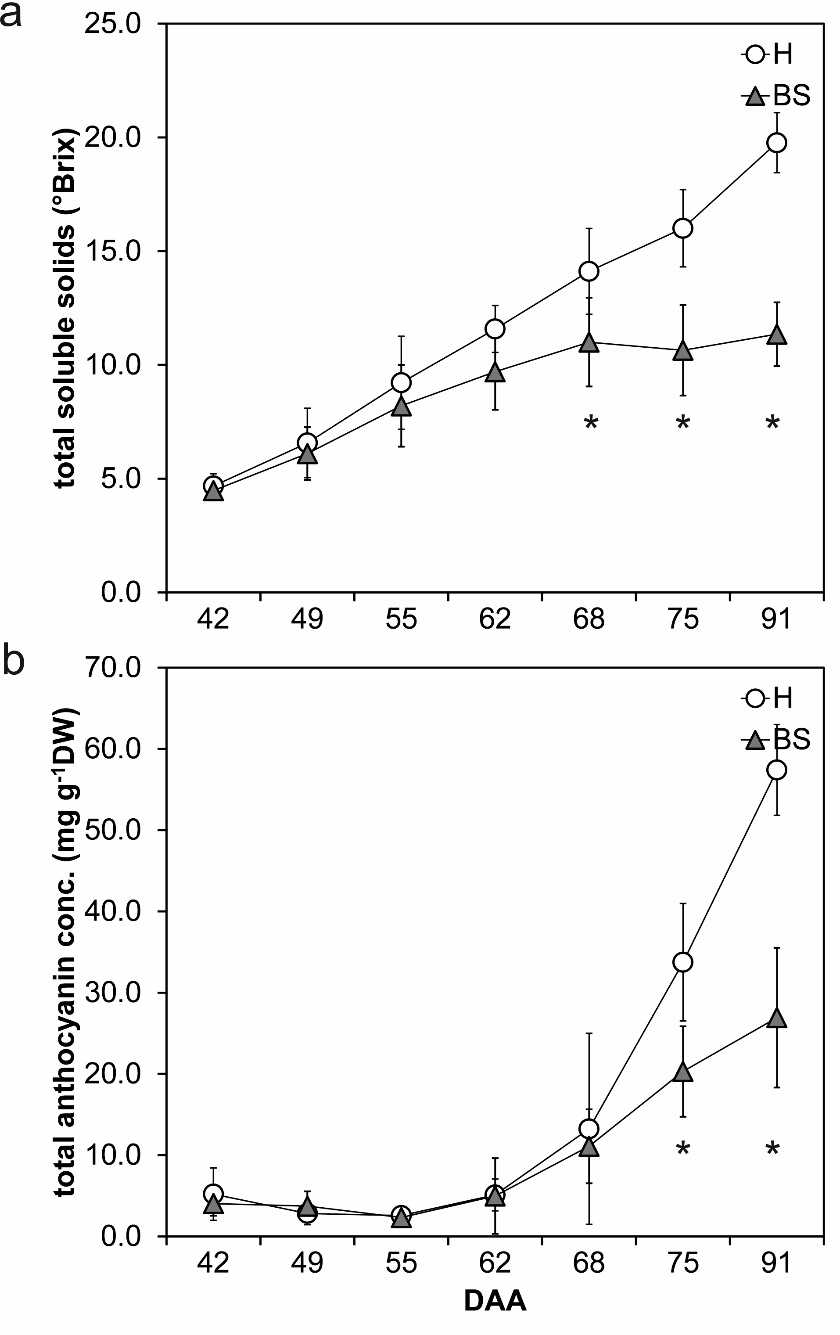


**Fig. S3** (**a**) Timescale of soluble solids (°Brix) measurement in the vineyard of 300 grape clusters and (**b**) total anthocyanin content of healthy (H) and berry shrivel (BS) grapes at 6 sampling dates. Data presented are mean values on dry weight basis ± standard deviation (*n*=4 each). Veraison is indicated in the figure (V) and the first symptomatic grape clusters were identified on 11.08.2011 (BS). Asterix indicates statistical significant differences obtained by Mann-Whitney U test (*P*<0.05) between healthy and BS samples.

**Table S1** List of genes and primers used for qPCR analyses

| **Gene** | **code** | **accession number** | **forward 5´-3´** | **reverse 5´-3´** | **product length (bp)** |
| --- | --- | --- | --- | --- | --- |
| Actin (ACT7) reference gene | *VviACT* | **VIT_04s0044g00580** | TGTGCTTAGTGGTGGGTCAA | ATCTGCTGGAAGGTGCTGAG | 174 |
| Ubiquitin A-52 | *VviUBI* | **VIT_16s0098g01190** | TTGATGCAATTGGCTAGGAA | TGTAACACTGCATGCACCAA | 185 |
| Phenylalanine ammonia-lyase 2 (PAL2) | *VviPAL2* | **VIT_13s0019g04460** | AATCTGTCGGGTGGCCGCAAT | TGTGTTGCTGCGCTCTGGAC | 136 |
| Chalcone flavonone isomerase | *VviCHI* | **VIT_13s0067g03820** | ACGCTCGCCGTCAAGTGGAA | GCGACCCGTCAAAGGCAAAATCG | 132 |
| Chalcone synthase 1 | *VviCHS1* | **VIT_14s0068g00930** | ATGATGTACCAACAGGGCTGC | CAGCTGTGATTTCAGAGCAGAC | 115 |
| Flavonone- 3-hydroxylase | *VviF3H1* | **VIT_04s0023g03370 VIT_18s0001g14310** | TTATCTGAGCAATGGGAGGTTCA | GCTCATCTTCCTCCTGTACATCT | 196 |
| Flavonoid-3'-hydroxylase | *VviF3´H* | **VIT_17s0000g07200** | TGGTGGTGGAGCTGATGGTCCT | AGCGTCAAACCGAGCATGGAGC | 125 |
| Dihydroflavonol 4-reductase | *VviDFR* | **VIT_18s0001g12800** | CTGAGCAAGCTGCATGGAAG | CAGTGATCGGGGAAAGAGCA | 135 |
| Leucoanthocyanidin dioxgenase | *VviLDOX* | **VIT_02s0025g04720** | ACCGTGTTAAGGTTGCTGGA | GTCCTCCCACTCAAGCTGTC | 146 |
| Leucoanthocyanidin reductase 1 | *VviLAR1* | **VIT_01s0011g02960** | TCTATTGATGGCCCGGAGGA | GGTCGGCTGCTTTTCCTCTA | 117 |
| Leucoanthocyanidin reductase 2 | *VviLAR2* | **VIT_17s0000g04150** | TCGCTTCATTTCCGACCTCC | TTCTTCCACGGTTACACGGG | 105 |
| Myb VvMYBA1 | *VviMYBA1/2* | **VIT_02s0033g00410** | TAGTCACCACTTCAAAAAGG | GAATGTGTTTGGGGTTTATC | 145 |
| MYBPA1 protein | *VviMYBPA1* | **VIT_15s0046g00170** | TATTGGGGTTGACGGGGTTG | TCGCTCAAGCAGTTGCAGAT | 151 |
| UDP-glucose:flavonoid 3-O-glucosyltransferase | *VviUFGT* | **VIT_16s0039g02230** | CCTAAGGGACAAGGCAAGGG | CCCAACTGCCTCATGTGCTA | 118 |
| Cell wall apoplastic invertase | *VviCWI* | **VIT_09s0002g02320** | AACCCACCAGCCTTACAGAA | CTGACCAGCAGCCATTGATA | 242 |
| Neutral invertase/beta fructofuronosidase | *VviCINV* | **VIT_05s0077g00510** | TTCATAGGCAAGCAGTCACG | TAGTCCTCTTCCCAAGCCAA | 116 |
| Vacuolar invertase 1 GIN1 | *VviGIN1* | **VIT_16s0022g00670** | GAGGGAAGAGGGGTGGCTCAGG | CAGGCAAACATGGCGGTAGTCCAA | 98 |
| Vacuolar invertase 2 GIN2 | *VviGIN2* | **VIT_02s0154g00090** | ACGCCTCACTTGTGTTTTCA | CAACGAGGTTTCCAACGG | 191 |
| Hexose transporter 1 | *VviHT1* | **VIT_00s0181g00010** | GAATTTCTGGTGGGGTCACGTCCAT | AGGCCACCAGCGACGAGAGA | 177 |
| Hexose transporter 7 (VvHT3, VvHT7) | *VviHT3/7* | **VIT_11s0149g00050** | GCGGGCCGAAGAAGACCACTAC | CGACCCGAAAGAAGCATCGCCA | 219 |
| tonoplast monosaccharide transporter | *VviTMT1* | **VIT_18s0122g00850** | CGGTGATGCTGACTTCAATCGGGT | ACCTGCCTTGCTCCAACTGCAAA | 157 |
| tonoplast monosaccharide transporter | *VviTMT2* | **VIT_03s0038g03940** | TCTTTCCCACCCGTGTCCGAGG | GCCAAAGACACCAGCAAGGCCA | 128 |
| tonoplast monosaccharide transporter | *VviTMT3* | **VIT_07s0031g02270** | CCAAGGGTGGCAAGGACAAGCA | CCACCCCAACAAACAATGCACGC | 129 |

**Table S2** Results of specific analyses of polyphenols from grape berry samples collected during the ripening phase (24.08.2011). BS symptoms were already visible. Data presented are mean values on dry weight basis ± standard deviation (*n*=4 each). Statistical significant differences (*P*<0.05) between healthy and BS samples are indicated with different letters. Compounds below the detection limit are shown as not detected (n.d.).

|  |  |  | **Contents (mean ± standard deviation)** | |
| --- | --- | --- | --- | --- |
| **Polyphenols determined by LC-MS** | **Classification** | **Unit** | **Healthy** | **Berry Shrivel** |
| caftaric acid | phenolic acid | µg g^-1^ berry DW | b 1772.0 ± 278.0 | a 4496.8 ± 335.8 |
| 4-coumaric acid | phenolic acid | µg g^-1^ berry DW | n.d. | n.d. |
| caffeic acid | phenolic acid | µg g^-1^ berry DW | n.d. | n.d. |
| ferulic acid | phenolic acid | µg g^-1^ berry DW | n.d. | n.d. |
| *trans*-resveratrol | stilbene | µg g^-1^ berry DW | n.d. | n.d. |
| *cis-*resveratrol | stilbene | µg g^-1^ berry DW | n.d. | n.d. |
| *trans*-resveratrol-3-*O*-glucoside | stilbene | µg g^-1^ berry DW | a 3.0 ± 1.8 | a 4.3 ± 1.1 |
| *cis*-resveratrol-3-*O*-glucoside | stilbene | µg g^-1^ berry DW | b 4.1 ± 1.0 | a 13.8 ± 4.4 |
| naringenin | flavanone | µg g^-1^ berry DW | n.d. | n.d. |
| eriodictyol | flavanone | µg g^-1^ berry DW | n.d. | n.d. |
| dihydroquercetin | flavanonol | µg g^-1^ berry DW | n.d. | n.d. |
| dihydrokaempferol | flavanonol | µg g^-1^ berry DW | n.d. | n.d. |
| dihydromyricetin | flavanonol | µg g^-1^ berry DW | a 15.9 ± 1.5 | a 18.1 ± 1.3 |
| quercetin | flavonol | µg g^-1^ berry DW | n.d. | n.d. |
| quercetin-3-*O*-glucuronide | flavonol | µg g^-1^ berry DW | b 141.9 ± 18.4 | a 442.9 ± 83.8 |
| quercetin-3-*O*-glucoside | flavonol | µg g^-1^ berry DW | a 102.5 ± 72.3 | a 122.8 ± 71.5 |
| kaempferol-3-*O*-glucoside | flavonol | µg g^-1^ berry DW | a 21.3 ± 18.4 | a 33.8 ± 36.2 |
| kaempferol | flavonol | µg g^-1^ berry DW | n.d. | n.d. |
| (+)-catechin | flavan-3-ol | µg g^-1^ berry DW | b 85.0 ± 47.4 | a 200.4 ± 42.7 |
| (-)-epicatechin | flavan-3-ol | µg g^-1^ berry DW | a 132.6 ± 138.4 | a 224.9 ± 145.9 |
| (-)-epicatechin gallate | flavan-3-ol | µg g^-1^ berry DW | a 15.4 ± 10.9 | a 32.1 ± 15.4 |
| delphinidin | anthocyanin/anthocyanidin | area *10^6^ g^-1^ berry DW | n.d. | n.d. |
| DEL-3-*O*-glucoside | anthocyanin/anthocyanidin | area *10^6^ g^-1^ berry DW | a 1391 ± 123 | b 498 ± 368 |
| DEL-3-*O*-(6-*O*-acetyl)-5-*O*-diglucoside | anthocyanin/anthocyanidin | area *10^6^ g^-1^ berry DW | a 0.6 ± 0.5 | a 0.7 ± 0.4 |
| DEL-3-*O*-(6-*O*-p-coumaroyl)-5-*O*-diglucoside | anthocyanin/anthocyanidin | area *10^6^ g^-1^ berry DW | a 262 ± 29 | b 47 ± 32 |
| cis-DEL-3-*O*-(6-*O*--coumaroyl)-glucoside | anthocyanin/anthocyanidin | area *10^6^ g^-1^ berry DW | a 413 ± 49 | a 272 ± 122 |
| trans-DEL-3-*O*-(6-*O*-p-coumaroyl)-glucoside | anthocyanin/anthocyanidin | area *10^6^ g^-1^ berry DW | b 10 ± 2 | a 23 ± 7 |
| cyanidin | anthocyanin/anthocyanidin | area *10^6^ g^-1^ berry DW | n.d. | n.d. |
| CYA-3-*O*-glucoside | anthocyanin/anthocyanidin | area *10^6^ g^-1^ berry DW | a 3.4 ± 1.3 | b 0.9 ± 0.5 |
| CYA-3-*O*-(6-*O*-p-coumaroyl)-5-*O*-(Ac)-diglucoside | anthocyanin/anthocyanidin | area *10^6^ g^-1^ berry DW | a 33 ± 9 | b 7 ± 3 |
| cis-CYA-3-*O*-(6-P-p-coumaroyl)-glucoside | anthocyanin/anthocyanidin | area *10^6^ g^-1^ berry DW | a 94 ± 30 | a 56 ± 20 |
| trans-CYA-3-*O*-(6-*O*-p-coumaroyl)-glucoside | anthocyanin/anthocyanidin | area *10^6^ g^-1^ berry DW | a 14 ± 4 | a 26 ± 14 |

**Table S3** Data obtained from relative gene expression (qPCR) calculated as NRQs of nine sugar related genes and thirteen polyphenol biosynthesis genes. Healthy and BS berries at six sampling dates were analyzed. Data shown are mean values ± standard deviation (*n*=4 each). Asterix indicates significant differences between healthy and BS grapes (*P*<0.05) as determined with Mann-Whitney U tests.

| **Relative gene expression sugar metabolism and anthocyanin biosynthesis. Normalized relative quantities (mean values ± standard deviation)** | | | | | | | | | | | | |
| --- | --- | --- | --- | --- | --- | --- | --- | --- | --- | --- | --- | --- |
| 1. **Sugar metabolism** | | | | | | | | | | | | |
|  | 22.07.^#^ | | 29.07.^#^ | | 04.08.^#^ | | 11.08.^#^ | | 17.08.^#^ | | 24.08.^#^ | |
|  | Healthy | BS | Healthy | BS | Healthy | BS | Healthy | BS | Healthy | BS | Healthy | BS |
| *VviHT1* | 3.80 ± 2.0 | 3.55 ± 1.4 | 0.91 ± 0.2 | 0.77 ± 0.4 | 2.66 ± 1.6 | 3.48 ± 1.7 | 1.16 ± 0.3 | 1.22 ± 0.3 | 0.79 ± 0.5 | 1.00 ± 0.5 | 0.48 ± 0.2 | * 1.51 ± 0.6 |
| *VviHT3* | 0.67 ± 0.2 | 0.55 ± 0.2 | 0.78 ± 0.2 | 0.75 ± 0.3 | 2.08 ± 1.1 | 4.50 ± 1.3 | 0.97 ± 0.2 | 1.01 ± 0.3 | 1.13 ± 0.3 | 1.38 ± 0.5 | 1.01 ± 0.1 | 1.03 ± 0.3 |
| *VviCINV1* | 0.85 ± 0.2 | 0.97 ± 0.1 | 0.59 ± 0.1 | 0.51 ± 0.1 | 0.76 ± 0.2 | * 2.38 ± 0.8 | 0.91 ± 0.2 | 1.34 ± 0.4 | 1.34 ± 0.6 | 1.47 ± 0.3 | 0.98 ± 0.1 | * 1.78 ± 0.1 |
| *VviGIN1* | 4.87 ± 0.8 | * 2.25 ± 0.7 | 2.31 ± 0.8 | 1.71 ± 0.8 | 3.60 ± 2.5 | 3.40 ± 1.4 | 2.81 ± 1.7 | 2.79 ± 0.7 | 0.63 ± 0.5 | 1.32 ± 1.0 | 0.61 ± 0.2 | 0.78 ± 0.1 |
| *VviGIN2* | 4.81 ± 1.1 | 5.65 ± 1.1 | 2.23 ± 0.3 | 2.29 ± 0.6 | 1.24 ± 0.2 | * 2.26 ± 0.6 | 1.38 ± 0.1 | * 2.22 ± 0.5 | 0.92 ± 0.4 | 0.45 ± 0.1 | 0.33 ± 0.1 | * 0.43 ± 0.1 |
| *VviCWInv* | 5.50 ± 0.9 | 3.71 ± 1.1 | 1.87 ± 0.5 | 3.24 ± 1.3 | 1.12 ± 0.4 | 1.00 ± 0.4 | 0.88 ± 0.2 | 0.54 ± 0.3 | 0.90 ± 0.4 | 1.40 ± 0.6 | 0.84 ± 0.2 | 0.43 ± 0.3 |
| *VviTMT1* | 1.99 ± 1.0 | 1.91 ± 0.8 | 3.47 ± 2.1 | 2.61 ± 0.7 | 5.59 ± 1.9 | 5.17 ± 1.0 | 6.12 ± 1.0 | 5.10 ± 0.9 | 6.65 ± 1.6 | 5.60 ± 0.6 | 3.78 ± 1.0 | 2.74 ± 0.2 |
| *VviTMT2* | 0.78 ± 0.2 | * 0.42 ± 0.1 | 0.64 ± 0.2 | 0.82 ± 0.3 | 1.24 ± 0.9 | 1.81 ± 0.6 | 0.76 ± 0.2 | 0.39 ± 0.1 | 1.78 ± 0.2 | 1.37 ± 0.6 | 4.74 ± 2.1 | * 1.54 ± 0.3 |
| *VviTMT3* | 1.64 ± 0.3 | * 0.76 ± 0.3 | 1.33 ± 0.6 | 1.42 ± 0.3 | 1.65 ± 1.0 | 1.91 ± 0.6 | 1.03 ± 0.5 | * 0.45 ± 0.2 | 1.01 ± 0.3 | 1.80 ± 0.6 | 0.59 ± 0.1 | 0.63 ± 0.3 |
| 1. **Anthocyanin biosynthesis** | | | | | | | | | | | | |
| *VviPAL2* | 0.28 ± 0.1 | 0.26 ± 0.1 | 0.40 ± 0.1 | 0.39 ± 0.1 | 0.60 ± 0.5 | 0.48 ± 0.3 | 1.56 ± 0.3 | 1.71 ± 0.6 | 2.92 ± 0.1 | 4.55 ± 2.4 | 6.17 ± 2.1 | 3.91 ± 1.2 |
| *VviCHS1* | 0.95 ± 0.4 | 0.54 ± 0.3 | 0.26 ± 0.0 | 0.32 ± 0.2 | 0.42 ± 0.0 | 0.56 ± 0.2 | 1.70 ± 0.8 | 1.00 ± 0.3 | 2.87 ± 1.5 | 1.97 ± 1.1 | 8.66 ± 2.5 | 5.26 ± 2.1 |
| *VviCHI1* | 0.55 ± 0.2 | 0.37 ± 0.2 | 0.27 ± 0.1 | 0.32 ± 0.1 | 0.92 ± 0.5 | 0.65 ± 0.4 | 1.55 ± 0.5 | 1.18 ± 0.4 | 2.15 ± 0.1 | 3.65 ± 1.8 | 5.11 ± 1.0 | * 2.47 ± 0.6 |
| *VviF3H* | 0.58 ± 0.2 | 0.66 ± 0.1 | 0.33 ± 0.0 | 0.40 ± 0.1 | 0.69 ± 0.1 | 0.92 ± 0.3 | 1.33 ± 0.3 | 1.18 ± 0.5 | 2.45 ± 1.0 | 2.50 ± 0.7 | 3.15 ± 0.3 | * 1.64 ± 0.4 |
| *VviF3'H* | 1.02 ± 0.3 | 0.63 ± 0.2 | 0.65 ± 0.1 | 0.70 ± 0.2 | 0.65 ± 0.1 | 0.83 ± 0.4 | 1.11 ± 0.6 | 0.61 ± 0.1 | 1.31 ± 0.4 | 1.64 ± 0.3 | 3.09 ± 1.1 | 2.14 ± 0.2 |
| *VviDFR* | 0.56 ± 0.1 | 0.55 ± 0.1 | 0.55 ± 0.1 | 0.55 ± 0.1 | 0.87 ± 0.1 | * 1.03 ± 0.1 | 1.59 ± 0.5 | 1.33 ± 0.5 | 1.53 ± 0.5 | 2.22 ± 0.7 | 1.85 ± 0.3 | 1.17 ± 0.2 |
| *VviLAR1* | 4.39 ± 0.9 | * 1.93 ± 1.2 | 0.49 ± 0.1 | 0.61 ± 0.3 | 2.35 ± 0.5 | * 1.05 ± 0.4 | 2.16 ± 0.4 | 2.04 ± 1.2 | 0.95 ± 0.4 | 0.87 ± 0.4 | 0.41 ± 0.1 | 0.30 ± 0.2 |
| *VviLAR2* | 4.76 ± 1.2 | 3.17 ± 1.9 | 1.95 ± 0.2 | 2.48 ± 1.2 | 0.51 ± 0.3 | * 0.91 ± 0.1 | 0.76 ± 0.7 | 0.64 ± 0.3 | 0.41 ± 0.1 | 0.48 ± 0.4 | 0.64 ± 0.1 | 0.70 ± 0.2 |
| *VviLDOX* | 0.42 ± 0.1 | 0.42 ± 0.2 | 0.49 ± 0.1 | 0.57 ± 0.1 | 0.64 ± 0.3 | * 1.19 ± 0.3 | 1.21 ± 0.3 | 0.92 ± 0.2 | 2.16 ± 0.4 | 2.55 ± 0.5 | 2.86 ± 0.6 | 2.11 ± 0.8 |
| *VviANR* | 8.03 ± 3.4 | 6.26 ± 1.5 | 1.53 ± 0.4 | 1.92 ± 0.8 | 0.41 ± 0.1 | * 0.68 ± 0.1 | 0.51 ± 0.1 | 0.45 ± 0.2 | 0.33 ± 0.0 | 0.49 ± 0.2 | 0.79 ± 0.2 | 0.54 ± 0.2 |
| *VviUFGT* | 0.18 ± 0.1 | * 0.02 ± 0.0 | 0.12 ± 0.1 | 0.01 ± 0.0 | 2.97 ± 0.6 | * 0.37 ± 0.3 | 5.02 ± 1.5 | 3.45 ± 1.0 | 14.64 ± 5.0 | 14.86 ± 5.3 | 13.90 ± 3.0 | 15.79 ± 6.7 |
| *VviMYBPA1* | 2.72 ± 1.3 | 2.27 ± 1.1 | 0.49 ± 0.2 | 1.12 ± 0.5 | 2.59 ± 0.3 | * 0.95 ± 0.5 | 0.71 ± 0.1 | 0.45 ± 0.2 | 1.79 ± 2.0 | 1.16 ± 0.9 | 1.77 ± 1.0 | 0.57 ± 0.3 |
| *VviMYBA1\2* | 0.65 ± 0.5 | 0.02 ± 0.0 | 0.32 ± 0.4 | 0.04 ± 0.0 | 1.71 ± 0.2 | * 0.47 ± 0.2 | 2.63 ± 0.8 | 3.79 ± 0.8 | 6.03 ± 1.7 | 8.60 ± 3.7 | 8.27 ± 2.9 | 11.37 ± 3.7 |
| ^#^ 22.July 2011 (BBCH 75), 29. July 2011 (BBCH 77-79), 4.August 2011 (BBCH 81), 11.August 2011 (BBCH 83), 17.August 2011 (BBCH85), 24.August 2011 (BBCH83-89) | | | | | | | | | | | | |
